# Chikungunya virus infection: molecular biology, clinical characteristics, and epidemiology in Asian countries

**DOI:** 10.1186/s12929-021-00778-8

**Published:** 2021-12-02

**Authors:** Sarawut Khongwichit, Jira Chansaenroj, Chintana Chirathaworn, Yong Poovorawan

**Affiliations:** 1 Center of Excellence in Clinical Virology, Department of Pediatrics, Faculty of Medicine, Chulalongkorn University, Bangkok, 10330 Thailand; 2 Department of Microbiology, Faculty of Medicine, Chulalongkorn University, Bangkok, 10330 Thailand; 3 Tropical Medicine Cluster, Chulalongkorn University, Bangkok, 10330 Thailand

**Keywords:** Chikungunya virus, Outbreak, Asia, Novel ECSA, E1: K211E and E2: V264A

## Abstract

Chikungunya virus (CHIKV) is a re-emerging mosquito-borne human pathogen that causes chikungunya fever, which is typically accompanied by severe joint pain. In Asia, serological evidence indicated that CHIKV first emerged in 1954. From the 1950’s to 2005, sporadic CHIKV infections were attributed to the Asian genotype. However, the massive outbreak of CHIKV in India and the Southwest Indian Ocean Islands in 2005 has since raised chikungunya as a worldwide public health concern. The virus is spreading globally, but mostly in tropical and subtropical regions, particularly in South and Southeast Asia. The emergence of the CHIKV East/Central/South African genotype-Indian Ocean lineage (ECSA-IOL) has caused large outbreaks in South and Southeast Asia affected more than a million people over a decade. Notably, the massive CHIKV outbreaks before 2016 and the more recent outbreak in Asia were driven by distinct ECSA lineages. The first significant CHIKV ECSA strains harbored the *Aedes albopictus*-adaptive mutation E1: A226V. More recently, another mass CHIKV ECSA outbreak in Asia started in India and spread beyond South and Southeast Asia to Kenya and Italy. This virus lacked the E1: A226V mutation but instead harbored two novel mutations (E1: K211E and E2: V264A) in an E1: 226A background, which enhanced its fitness in *Aedes aegypti*. The emergence of a novel ECSA strain may lead to a more widespread geographical distribution of CHIKV in the future. This review summarizes the current CHIKV situation in Asian countries and provides a general overview of the molecular virology, disease manifestation, diagnosis, prevalence, genotype distribution, evolutionary relationships, and epidemiology of CHIKV infection in Asian countries over the past 65 years. This knowledge is essential in guiding the epidemiological study, control, prevention of future CHIKV outbreaks, and the development of new vaccines and antivirals targeting CHIKV.

## Background

**Fig. 1:**
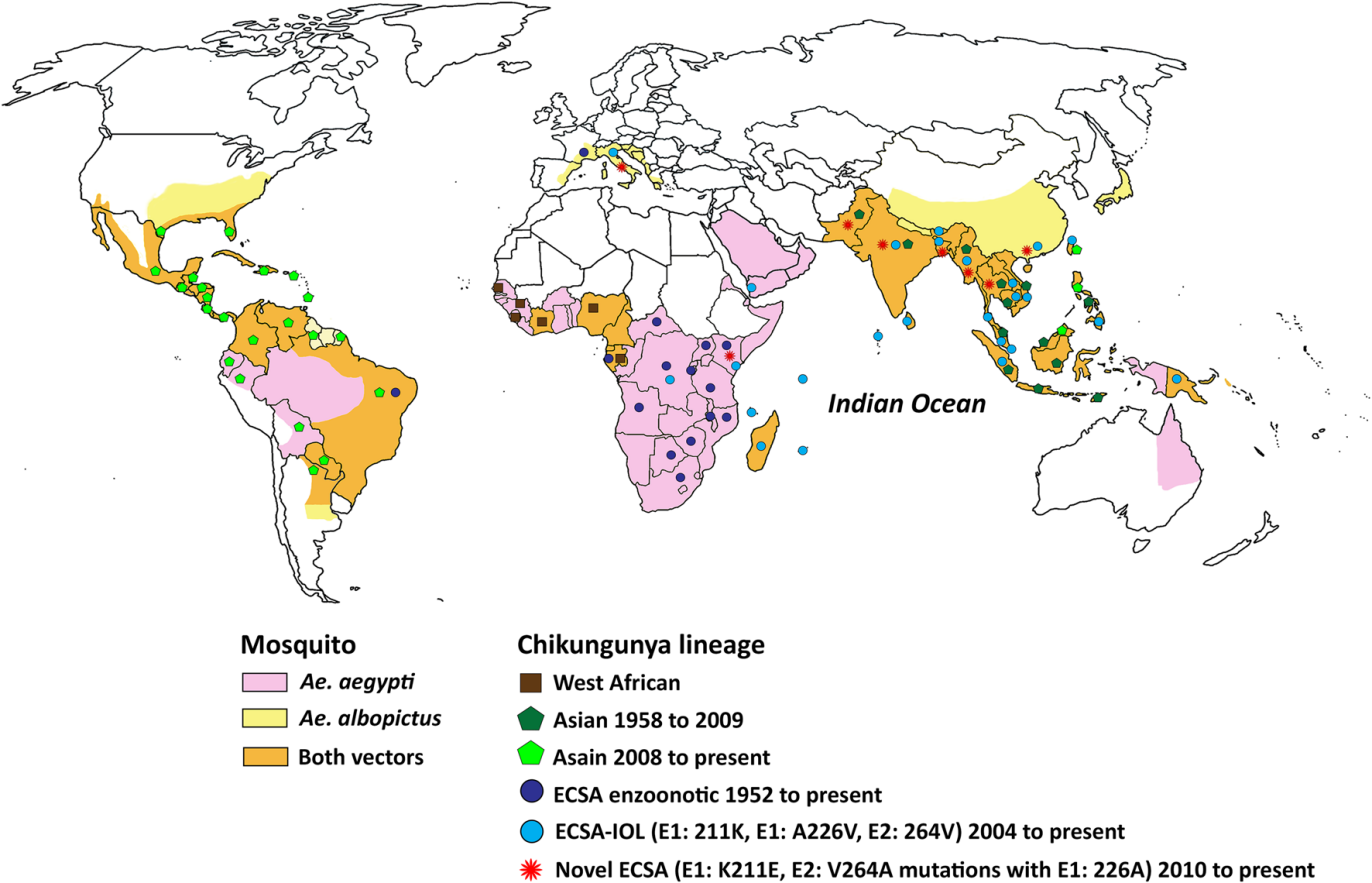
Geographical distribution of CHIKV lineages during 1952–2020 and that of its primary mosquito vectors. Different CHIKV lineages are represented by distinct colored symbols. The map also shows the distribution of the primary CHIKV mosquito vectors. Areas shown in pink indicate the range of *Ae. aegypti*, those shown in yellow indicate the range of *Ae. albopictus*, and the range of both primary vectors is indicated in orange. The range of both primary vectors was obtained from [16]. Distribution of chikungunya virus data were obtained from a number of studies [9, 17–51]

**Fig. 2:**
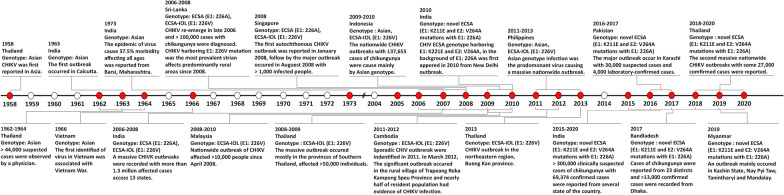
Historical timeline of major CHIKV outbreaks in Asia (1958–2020)

Chikungunya virus (CHIKV) is a re-emerging mosquito-borne pathogen classified as a member of the *Alphavirus* genus in the family *Togaviridae* [1]. Infection by CHIKV typically results in mild and self-limiting disease in infected humans, characterized by fever, skin rash, myalgia, and arthralgia that can last weeks to months [2, 3]. Symptoms usually appear 4–7 days after exposure to CHIKV [4]. Although chikungunya fever is a self-limiting disease and the associated fatality rate is low, chikungunya-related death has been reported in young infants, the elderly, and people with pre-existing conditions such as cardiovascular disease, diabetes, kidney disease, and chronic liver disease [5–8]. An atypical clinical manifestation of CHIKV infection was associated with an increased mortality rate during the last chikungunya outbreak on the island of Réunion in the Indian Ocean in 2005–2006 [5]. This mass outbreak was associated with the CHIKV East/Central/South African (ECSA) genotype harboring mosquito vector-adaptive mutations [9]. The adapted virus has subsequently threatened to undergo both endemic and epidemic spread in Africa, Asia, Europe, and America [10]. Ten years later, novel mutations in CHIKV were reported to have facilitated the second mass outbreak in South and Southeast Asia (Figs. 1 and 2) [11–15]. Here, we summarize the available reported data for CHIKV circulating in South and Southeast Asia and the available information on the history of CHIKV epidemiology, its molecular biology, clinical features, and genotypic distribution. This knowledge is important for understanding the epidemiology of CHIKV, and may lead to the development of novel approaches for controlling and preventing future outbreaks and guiding future research on the mechanisms underlying viral infections, as well as the development of new vaccines and antivirals targeting CHIKV.

## Main text

### The molecular structure and transmission cycle of CHIKV

CHIKV has an enveloped, 60–70-nm diameter, icosahedral nucleocapsid containing a positive-sense single-stranded RNA genome of approximately 11.8 kb. The genome comprises two open reading frames (ORFs), ORF1 and ORF2, flanked by a 5′ cap and a polyadenylated tail at the 3′UTR. These ORFs encode polyprotein precursors of four nonstructural (nsP1, nsP2, nsP3, and nsP4) and six structural proteins (capsid [C]; envelope proteins E1, E2, and E3; 6K; and transframe proteins [TM]), respectively [52, 53]. CHIKV has been categorized into three major lineages: West African, Asian, and East/Central/South/Africa (ECSA). The Indian Ocean lineage (IOL) is a sub-lineage evolved from the ECSA lineage [21, 54]. A comprehensive phylogenetic study of historic CHIKV strains indicated that the virus originated in Africa and has episodically spread into Asia in approximately 50-year intervals [55]. The IOL sub-lineage or ECSA-IOL genotype was emerged in Kenya in 2004 and then spread to several islands in the Indian Ocean, the Indian subcontinent, and Southeast Asia [56, 57]. In nature, CHIKV has both a sylvatic and an urban transmission cycle. CHIKV originated in Africa through a sylvatic cycle involving transmission between wild, nonhuman primates and forest-dwelling *Aedes* spp. mosquitoes, such as *Ae. taylori*, *Ae. luteocephalus*, *Ae. furcifer*, *Ae. neoafricanus*, and *Ae. africanus* (animal–mosquito–animal) and was subsequently introduced into Asia [58–60]. Virus transmission is mainly maintained in an urban cycle from human to human, with transmission occurring primarily via the bite of infected *Ae. aegypti* or *Ae. albopictus* [61–64].

### CHIKV pathogenesis and cellular tropism

As noted previously, CHIKV infection is characterized by chronic and severe polyarthralgia and/or polyarthritis, which may persist for a week or several months. Polyarthralgia and/or polyarthritis can affect any joint but is most common in small joints, such as the ankles, wrists, and phalanges [65, 66]. Studies have shown that animals and human patients suffering from persistent polyarthralgia induced by CHIKV infection display strong immune responses against the virus, such as the infiltration of immune cells (e.g., macrophages, natural killer [NK] cells, and CD4^+^ T cells) into synovial tissues and increased secretion of pro-inflammatory mediators [67–69]. Elevated levels of interleukin 6 (IL-6) and granulocyte-macrophage colony‐stimulating factor (GM‐CSF) were shown to be correlated with an increased incidence of persistent joint pain, indicating that an association may exist between chronic arthritic symptoms and CHIKV infection [70].

CHIKV has broad cellular tropism and can infect a wide variety of cell lines and human primary cells in vitro, including human epithelial cell lines (Hela, HEK293, and HUH7), the human microglia cell line CHME5, human hepatocytes (HepG2 cells), a neuroblastoma cell line, primary fibroblasts (MRC5 lung cells), primary human skeletal muscle myoblasts (HSMMs), and monocyte-derived macrophages [71–75]. In nonhuman primates, macrophages were observed to serve as the primary cellular reservoirs of persistent CHIKV in the chronic phase of the disease [76].

### Cellular entry factors for CHIKV infection

The host cell entry and attachment factors for CHIKV have been identified and characterized in insect and mammalian cells. The first characterized entry factor was prohibitin (PHB), which was shown to mediate the internalization of the virus into human microglial CHME-5 cells [77]. A potential CHIKV entry factor was also identified in the mosquito. ATP synthase β subunit (ATPSβ) was found to interact with envelope protein 2 (E2) of CHIKV in mosquito cells [78]. Matrix-remodeling-associated protein 8 (MXRA8) was the third entry factor to be characterized. This cell adhesion molecule was found to play a role as an entry factor for CHIKV and other alphaviruses, including O’nyong-nyong virus (ONNV), Ross River virus (RRV), and Mayaro virus (MAYV) [79–81]. Mice with Mxra8 gene defect show reduced levels of CHIKV infection, replication, and pro-inflammatory cytokines, including IL-6, MIP-1β, GM-CSF, and G-CSF [81]. However, residual CHIKV infection in mice lacking Mxra8, and the absence of a mosquito orthologue of Mxra8, indicate that additional as yet undiscovered host proteins mediate CHIKV entry into host cells. Some cell surface components, such as glycosaminoglycans (GAGs), serve as attachment factors for CHIKV [82–85]. Many pathogenic viruses utilize GAGs as attachment factors [82–84, 86–92]. Point mutations in domain A of the CHIKV E2 protein such as E79K, G82R, or E166K have been identified and exhibit enhanced GAG dependency but decreased in vivo replication [82, 83, 93]. These alterations influence the binding affinity of the virus by increasing the positive charge in domain A of the E2 protein [83]. Although GAGs promote CHIKV entry and replication, these cell surface proteins are not certainly required for infection, as CHIKV can still enter GAG-deficient cells [84]. Numerous studies have shown that phosphatidylserine-binding proteins, such as T-cell immunoglobulin and mucin (TIM)-1 and TIM-4, function as a CHIKV attachment factors, enhancing CHIKV binding and entry into host cells [94–96]. Similarly, dendritic cell-specific intercellular adhesion molecule 3-grabbing nonintegrin (DC-SIGN) is an essential factor for enhancing the infectivity of alphaviruses such as CHIKV and Semliki Forest virus [97]. Additionally, DC-SIGN or CD209 gene polymorphisms appear to be associated with the clinical progression of chikungunya fever [98]. Furthermore, previous study have identified the interaction host protein partner of CHIKV E2 protein using a yeast two-hybrid assay with human brain cDNA library. Three candidate E2-binding protein partners were discovered, including actin, gamma 1 (ACTG1), protein tyrosine phosphatase, non-receptor type 2 (PTPN2), and collagen type 1 alpha 2 (COL1A2) [99]. Nevertheless, the precise roles played by ACTG1, PTPN2, and COL1A2 in CHIKV infection remain unclear. Although several proteins have been characterized as factors mediating the entry of CHIKV into host cells, interfering with the genes coding for these CHIKV entry factors does not fully inhibit viral infection and production, indicating that CHIKV employs multiple mechanisms to invade the host cells [100].

### Clinical symptoms and signs

In Makonde, chikungunya means “that which bends up,” and refers to the bent posture of CHIKV-infected patients with severe arthralgia [101]. Based on symptom duration, chikungunya infection is classified into three stages, namely, an acute stage (from around 3–7 days after exposure to the virus until the end of day 21), a post-acute stage (after the first 3 weeks until the 3rd month after onset), and a chronic stage, which starts from 3 months after disease onset. After an incubation period of 3–7 days following a bite from a CHIKV-infected mosquito, most CHIKV infections become symptomatic, with the most common symptoms being acute febrile illness (> 38.9 °C), arthralgia, rash, and headache [102, 103]. Acute symptoms usually resolve within 7 to 14 days [104, 105]. However, not all infections exhibit symptoms. Serosurveys revealed that 3–25% of individuals infected with CHIKV show no symptoms [106–108].

Severe arthralgia or joint pain is the main manifestation of chikungunya. The joint pain is most often symmetrical and polyarticular, affecting both small and large peripheral joints such as the wrists, elbows, ankles, knees, hands, feet, and shoulders [103]. CHIKV infection can lead to severe joint pain with or without arthritis and last for several months to years in the chronic stage [65, 66]. Persistent arthralgia in chikungunya is frequently debilitating, resulting in an impaired quality of life [109, 110]. The prevalence of chikungunya-associated patients progressing to a chronic stage has been reported to range between 4.1 and 78.6%; however, the reasons for this variability remains unclear [111, 112]. The possibility of chronic consequence may be due to persistent virus infection, tissue injury caused by a viral infection, exacerbation of a pre-existing joint condition, and genetic susceptibility [113–115]. Even though chikungunya infection rarely progresses to a severe or life-threatening form, atypical clinical symptoms of chikungunya, such as cardiovascular and neurological manifestations, can promote a significant increase in morbidity. A wide variety of chikungunya-associated neurological manifestations have been reported, such as encephalitis, myelopathy, peripheral neuropathy, myelitis, and meningoencephalitis [116–119]. Death from chikungunya disease is infrequent and results mainly due to existing health problems or severe clinical manifestation in the elderly, infants, or immunocompromised patients [120–122].

### Diagnosis of CHIKV

As with other arbovirus infections, individuals infected with chikungunya can present a broad spectrum of symptoms, including high fever, headache, skin rash, myalgia, arthralgia, and neurological complications [3, 123]. The overlapping symptoms of chikungunya and other arbovirus diseases make diagnosis based on clinical symptoms alone challenging, particularly when CHIKV is co-circulating in the areas in which DENV and ZIKV are endemic [124, 125]. Thus specific laboratory diagnosis is necessary to validate the differential diagnosis of chikungunya and other etiologic agents of the disease [126]. The laboratory diagnosis of CHIKV infection is accomplished by examining the plasma or serum of suspected patients. Several methods are used to identify CHIKV, including viral isolation using cell culture and viral nucleic acid detection by reverse transcription-polymerase chain reaction (RT-PCR) and detection of CHIKV-specific IgM antibodies using a serological assay [127, 128]. The laboratory diagnostic testing algorithm to confirm CHIKV infection developed by the Center for Disease Control and Prevention (CDC) is dependent on disease characteristics and the timing of sample collection. RT-PCR can generally be used to detect viral RNA in the 1st week after the onset of symptoms, while an enzyme-linked immunosorbent assay (ELISA) is used as the serological test for the detection of CHIKV-specific IgM antibodies [129]. The sensitivity of detection of these antibodies in chikungunya patients increases for samples collected beginning after approximately 5 days of illness [130]. However, false-positive results can occur due to cross-reactivity with other arboviruses. In particular, antibody cross-reactions with DENV and the Semliki Forest antigenic complex group, such as Mayaro and o’nyong-nyong, have been observed [131–134]. Although viral culture is the gold standard for the diagnosis of CHIKV infection, the isolation of infectious CHIKV in cell culture from a patient’s blood is generally used as a research tool rather than for routine diagnostic purposes. Viral culture for CHIKV detection shows the greatest sensitivity in the viremia phase, typically within 5 days after disease onset [135].

### History of CHIKV emergence and re-emergence

The earliest detailed description of CHIKV appeared in 1952 during an outbreak in Tanzania. CHIKV was first isolated from a febrile patient in this country in early 1953 [136]. Since 1954, CHIKV has disseminated from its origin throughout Asian countries, including Thailand (1958), Cambodia (1961), Vietnam (1967), Myanmar (1975), Sri Lanka (1969), and India (1963) [137–142]. The virus has also been moving further east in the Pacific, including to the Philippines and Indonesia [143, 144]. In Asia, CHIKV was first isolated from the sera of patients in Bangkok, Thailand, in 1958, and this isolate was classified as an Asian genotype [138]. The first significant reported urban outbreak in Bangkok was recognized in the 1960s. During the period 1962–1964, rates of CHIKV infection in the Bangkok area were dramatically high, with an estimated 44,000 suspected cases of CHIKV infection, including two reports of encephalitis [145–149]. In India, the first CHIKV outbreak occurred in 1963 in Calcutta. Clinically, the patients presented with atypical symptoms, including hemorrhagic manifestations and neurological complications, with deaths also being documented. In the 1973 outbreak in India, the morbidity rate was reported to be 37.5% in Barsi, Maharashtra state. These CHIKV outbreaks were caused by the Asian genotype [150, 151]. Before 2000, CHIKV epidemics were mainly sporadic and limited to Africa and Asia; however, since 2000, CHIKV outbreaks have become more frequent. Extensive evidence, including molecular genetic evidence, indicates that CHIKV may have evolved adaptations to new mosquito vectors [9, 152]. Following the virus outbreak on the Kenyan coast between 2004 and 2005, the outbreak spread rapidly to several islands of the Indian Ocean, India, Sri Lanka and Southeast Asia, resulting in millions of infected people [152, 153]. During the mass outbreak on La Réunion in the southwestern part of the Indian Ocean, the surveillance system estimated 244,000 people (35% of the population) were affected by chikungunya, and high levels of excess deaths were associated with CHIKV infection [23, 154, 155]. Many studies have indicated that an *Ae. albopictus*-competent CHIKV strain was responsible for the mass chikungunya outbreak in La Réunion Island in 2005–2006 [156–158]. Remarkably, sequencing and evolutionary analysis of CHIKV isolates from La Réunion Island and other islands in the Indian Ocean suggested that the epidemic was associated with a strain of CHIKV harboring a single mutation in the viral envelope protein (E1: A226V) that was derived from the ECSA genotype (the IOL). This single amino acid substitution facilitated CHIKV transmission by *Ae. albopictus*, but not by *Ae. aegypti* [9, 57], indicating that *Ae. albopictus* was the primary transmission vector in the La Réunion outbreak. The number of epidemics associated with this vector subsequently increased worldwide, both locally and in new geographical areas (Africa, Europe, and Asia) where *Ae. albopictus* is widespread. However, local transmission was not detected in America until 2013; the etiologic agent was a strain of the Asian lineage [159].

#### India

The first recorded outbreak of CHIKV in India was reported in Kolkata, West Bengal, in 1963 [160, 161]. This was followed by a number of other outbreaks in Tamil Nadu, Andhra Pradesh, and Maharashtra during 1964–1965 [162]. The virus virtually disappeared from India after the last reported outbreak during the twentieth century was documented in Maharashtra in 1973 [150]. The circulating virus that caused outbreaks in India until the 1970s was reportedly the Asian genotype [163]. After a gap of 32-years, CHIKV re-emerged in the country and caused a massive outbreak between late 2005 and 2008. The causative CHIKV strain was of the ECSA lineage. More than 1.3 million cases of CHIKV infection were documented in almost every Indian state, including Karnataka, Madhya Pradesh, Andhra Pradesh, Tamil Nadu, and Maharashtra [36, 151, 164]. Phylogenetic analysis of CHIKV isolates obtained from 2006 demonstrated that the Indian CHIKV strain isolated in 2006 was more evolutionarily related to the viral strain from East Africa and the Indian Ocean islands but did not harbor the adaptive E1: A226V mutation [165]. Nevertheless, acquisition of the E1: A226V substitution was later observed in the southern Indian state of Kerala in 2007. In 2007, Kerala was the worst-affected state in the country, accounting for 55.8% of suspected chikungunya cases. High abundance of *A. albopictus* in the rubber plantations region of Kerala may have contributed significantly to the explosive spread of CHIKV in this state [28, 166, 167].

#### Sri Lanka

CHIKV re-emerged in October 2006 after a hiatus of 40 years. During 2006–2007, over 100,000 cases of CHIKV infection were diagnosed [168, 169]. The early chikungunya cases that arose predominantly in the dengue-endemic urban area and coastal towns during 2006–2007 were identified as an E1: 226A-carrying strain of the ECSA lineage. In 2008, CHIKV spread primarily in the rubber and banana plantation areas, which have high densities of *Ae. albopictus*. Most of the 2008 CHIKV Sri Lankan isolates possessed the E1: A226V substitution, which increased virus replication and dissemination in *Ae. albopictus*. Reports from Sri Lanka and Singapore also indicated that the strain responsible for the significant outbreaks in Sri Lanka, Singapore, and Malaysia were more genetically related to the Indian CHIKV isolates than to the Indian Ocean isolates [26].

#### Bangladesh

In Bangladesh, the first confirmed case of CHIKV infection in humans was documented in 2008 and was based on serologic evidence. The first recognized outbreak occurred in the northwest part of Bangladesh near the border with India [170]. In 2009, an epidemic of CHIKV with restricted geographic spread was detected in Santhia Upazila [171]. Other small-scale outbreaks of CHIKV were reported in rural areas in 2011 [172] and 2012 [173]. Phylogenetic analysis of the whole genome of CHIKV indicated that the CHIKV strain isolated in 2011 belonged to the ECSA-IOL genotype, forming a cluster with Indian CHIKV strains from 2009 [174]. Since then, reports of sporadic cases and small-scale outbreaks of CHIKV continued from 2013 to 2016 [171, 175].

#### Pakistan

CHIKV is likely to have been present for several decades as CHIKV-specific antibodies were identified in rodents and a few humans as long ago as 1983 [176]. In 2011, three cases of chikungunya were reported during the dengue virus outbreak in Lahore, Pakistan [177].

#### Malaysia

Malaysia has also experienced CHIKV outbreaks of both the Asian and ECSA genotypes. The first outbreak occurred in Port Klang, with more than 51 people being infected between December 1998 and February 1999 [178]. The Asian genotype was responsible for the first outbreak. The second CHIKV outbreak, which occurred between March and April 2006, affected over 200 people in Bagan Panchor. Notably, although this outbreak in Malaysia coincided with the global outbreak associated with the ECSA genotype, evidence showed that the second outbreak was driven by the endemic Asian lineage [179]. The third outbreak in December 2006 in Ipoh, Perak, resulted from the import of the ECSA genotype from India [180]. These first three outbreaks were relatively limited in scale, restricted as they were to a single area affecting approximately 300 people. Between April 2008 and 2010, a nationwide CHIKV outbreak was recorded in 14 out of 15 states and federal territories in Malaysia, with some 10,000 people affected. Sequence analysis revealed that an ECSA strain harboring the adaptive E1: A226V mutation was responsible for this extensive nationwide outbreak [27].

#### Singapore

In Singapore, although the country has experienced dengue transmission since 1960 [181], the first case of locally transmitted CHIKV infection was only reported in January 2008 [182]. A significant CHIKV outbreak occurred in Singapore in July 2008, leading to over 1000 infections, and was associated with the circulation of both the wild-type (226A) and mutant (A226V) E1-carrying CHIKV belonging to the ECSA. Phylogenetic tree analysis grouped CHIKV strains isolated from May to July 2008 with Malaysian isolates [183]. During 2010–2012, only sporadic cases were reported in Singapore. In 2013, Singapore again experienced a significant outbreak involving 1059 laboratory-confirmed cases of chikungunya infection [184].

#### Thailand

A significant outbreak in Thailand during 2008–2009 was also driven by CHIKV of the ECSA-IOL, resulting in the infection of approximately 54,000 individuals. The outbreak occurred in at least 58, mostly southern, provinces of Thailand, including Narathiwat, Phuket, Phatthalung, Songkhla, Pattani, Yala, Surat Thani, Phangnga, and Nakhon Si Thammarat [185, 186]. In 2010, a total of 1565 cases of CHIKV infection were reported in Thailand. In the ensuing 3 years, circulating CHIKV ECSA-IOL strains in the country caused an outbreak in Bueng Kan province in northeast Thailand [187]. Phylogenetic studies suggested that CHIKV strains isolated during the early and late 2008–2009 outbreak belonged to the ECSA genotype and were most closely related to CHIKV isolates detected in India in 2007 and Singapore in 2008 [188]. However, previously the predominant circulating strains of CHIKV in Thailand were of Asian genotype [21, 138, 188]. In addition, there is evidence that banked serum samples from five patients affected in 2009 also indicated the involvement of the Asian genotype of CHIKV. Among these samples, the E1 sequence of CHIKV isolated from patient serum collected from Songkhla Province, Thailand, exhibited 100% identity to the 2010 Indonesian isolate (CHIK/SBY8/10 isolate, GenBank Accession number AB678677) [189].

#### Myanmar

In Myanmar, all CHIKV strains isolated in 2010 from patients displaying dengue-like syndromes belonged to the ECSA-IOL genotype. The isolates were phylogenetically grouped with the 2008 Malaysia isolate, 2009 Thailand isolates, and 2010 China isolates [190].

#### Cambodia

CHIKV epidemic was reported in 1961, possibly as a result of the emergence of a CHIKV strain of Asian lineage [191]. In 2011, CHIKV of the ECSA-IOL emerged in Cambodia, with all the isolates from this outbreak being closely related to the strain that caused the outbreak in southern Thailand in 2008–2009 [192] and a small outbreak in Vietnam in 2012 [193].

#### Lao

Lao, which borders Cambodia, has reported sporadic CHIKV outbreaks in the provinces near the border between the two countries. Although the mosquito vectors *Ae. albopictus* and *Ae. aegypti* are present in the country, no cases of CHIKV infection were documented in Lao until 2012, when the first proven case of CHIKV was reported and resulted from the importation of the Cambodian strain of the virus [194, 195].

#### Vietnam

Chikungunya was first described in the 1960s [196]. The appearance of chikungunya was associated with the Vietnam War [140]. At that time, the most common genotype was the Asian genotype [22]. Although Vietnam borders countries where CHIKV outbreaks have occurred, including Thailand and Cambodia, only four sporadic cases (4/5,617; 0.07%) were identified in a retrospective study of serum samples collected between October 2010 and December 2014 from patients under 16 years of age with acute fever of less than 3 days. The four CHIKV isolates were of the IOL within the ECSA genotype, and were closely related to the 2011 Cambodian strain [193]. Further surveillance of CHIKV infection in patients hospitalized (N = 558) in Vietnam with acute febrile illness from September 2012 to September 2014 failed to identify cases of CHIKV infection. However, CHIKV was detected in two mosquitoes (*Ae. aegypti*) collected from Dac Nong and Long An province [197].

#### Republic of the Philippines

In the Philippines, CHIKV was first isolated in 1965 [198]. Sporadic cases were documented in Cebu, Masbate, and Mindanao islands in 1986 [199] and Cavite and Luzon in 1996 [107]. During 2011–2013, there was an extensive nationwide outbreak due mainly to the CHIKV Asian genotype. The co-circulation of the Asian genotype and the ECSA-IOL harboring E1: A226V was detected during 2011–2013. Nevertheless, the spread of the ECSA-IOL genotype was limited to the southern provinces of Mindanao. The transmission of both Asian and ECSA-IOL genotypes during the nationwide outbreak resulted from the movement of people near the country’s border [200].

#### Indonesia

According to official reports from the Ministry of Health of the Republic of Indonesia, chikungunya cases were formally reported for the first time in Samarinda, East Kalimantan, in 1973 [201, 202]. However, a serological survey of CHIKV infections demonstrated that the first occurrence of chikungunya was in 1964 [203]. In 1982, a CHIKV outbreak was virologically confirmed in Jambi, South Sumatra [144]. Subsequently, frequent CHIKV outbreaks were documented between 1982 and 1985 throughout the country [144, 204]. After a hiatus of nearly two decades, CHIKV re-emerged, and was responsible for at least 24 probable outbreaks throughout Indonesia from September 2001 to March 2003 [205]. In 2009–2010, there was a significant nationwide outbreak of the virus, during which 137,655 CHIKV people were infected. Based on sequencing and evolutionary analysis, most of the Indonesian isolates were characterized as Asian genotypes, and the rest as ECSA genotypes. The Indonesian ECSA genotype was first identified in May 2008. The Indonesian ECSA isolates in 2008 and 2011 appear to have been most closely related to the ECSA viruses that caused the massive outbreaks in Southeast Asian countries during the same period [206].

### The emergence of a novel sub-lineage of ECSA chikungunya virus

**Fig. 3:**
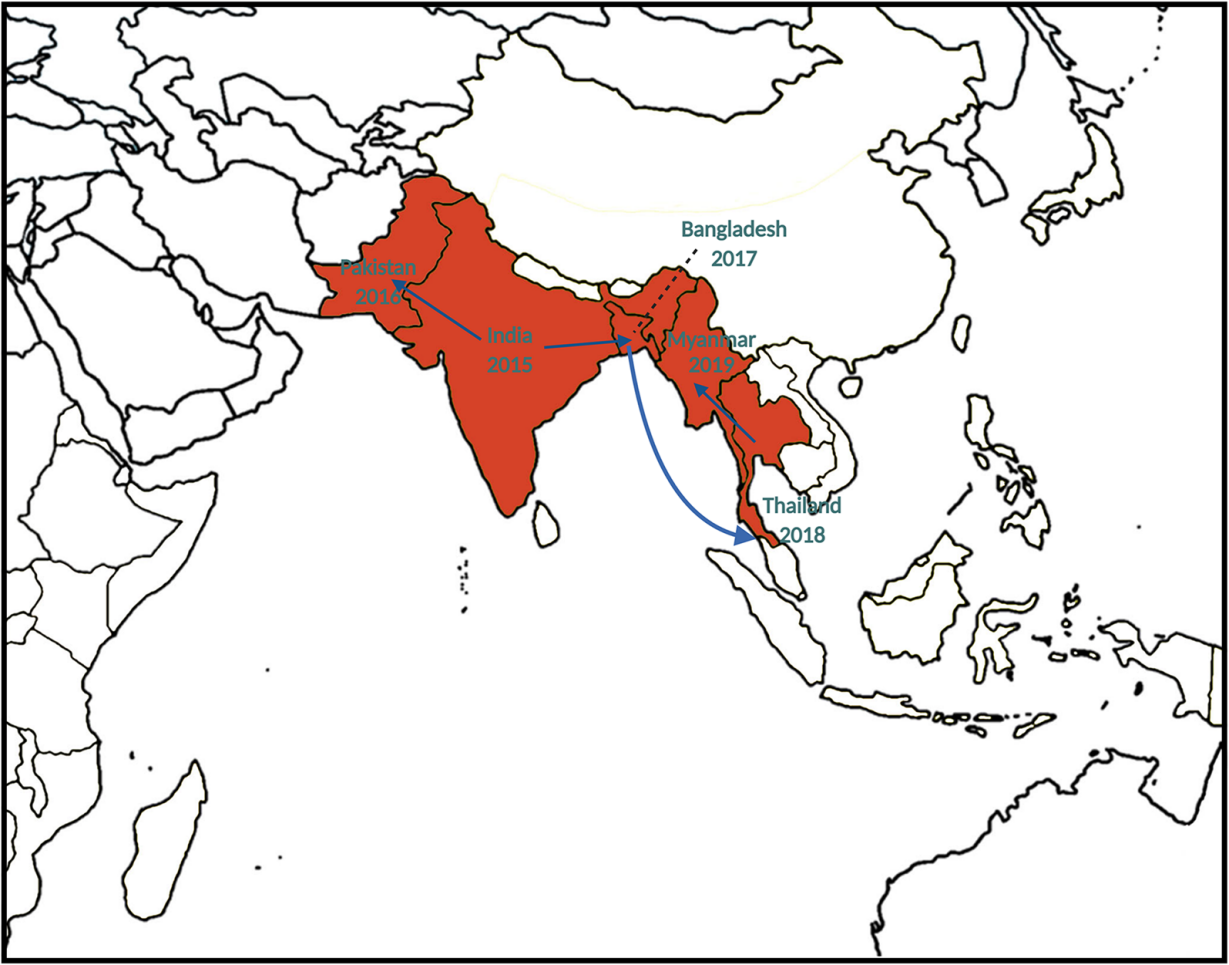
Distribution and spread of the novel sub-lineage of CHIKV East/Central/South African (ECSA) genotype in South and Southeast Asia during 2015–2019. *CHIKV* Chikungunya virus, *ECSA* East/Central/South African genotype

**Fig. 4:**
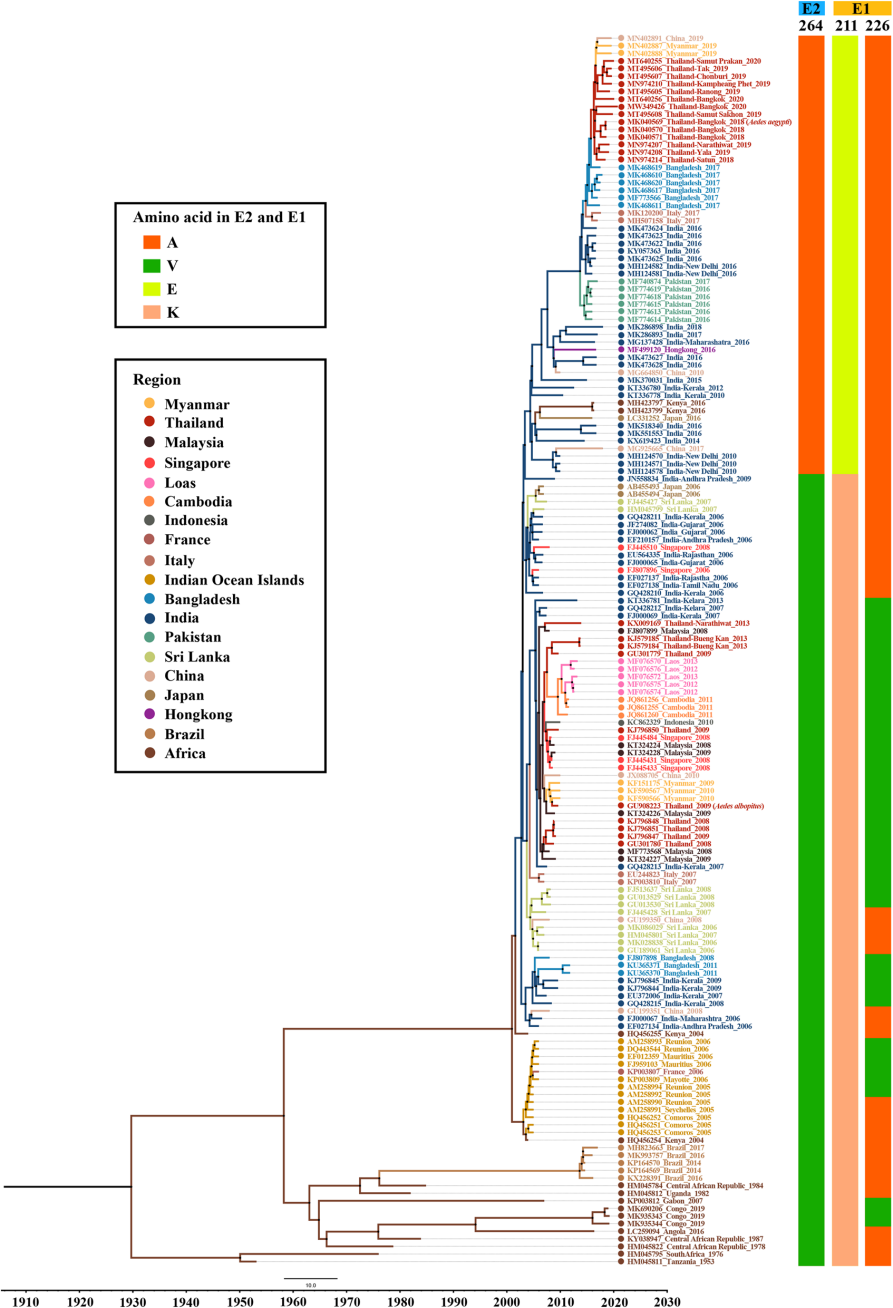
Evolutionary analysis of the complete genome of the CHIKV ECSA lineage. Bayesian time-scale tree of 169 genomes of the CHIKV ECSA lineage based on the strict clock model. The 95% HPD values of the most recent common ancestors (tMRC) are shown beside the nodes. The colored square of each strain corresponds to the amino acid mutations specific for the CHIKV ECSA lineage. *CHIKV* chikungunya virus, *ECSA* East/Central/South African, *HPD* highest posterior density

The CHIKV outbreaks that occurred in Asia between 1960 and 1999 were caused by Asian genotypes. Affected countries included India and Pakistan in South Asia and Thailand, Malaysia, Cambodia, Myanmar, Vietnam, Indonesia, and the Philippines in Southeast Asia [22]. Nevertheless, the explosive CHIKV outbreaks in the Indian Ocean Islands that have occurred since 2005 and the global increase in transportation have altered the distribution of circulating CHIKV genotypes. Different CHIKV ECSA lineages, especially the ECSA-IOL, have expanded locally and also spread to new regions of the Indian Ocean [207], Asia [41, 169, 183, 188, 208, 209], and Europe [24, 210]. Since 2016, the emergence of a unique CHIKV ECSA genotype-associated molecular signature has been reported from several parts of the world [12, 46, 211–213]. The genome of the new distinct CHIKV ECSA lineage contains two novel mutations—E1: K211E and E2: V264A—in a wild-type E1: 226A background. This lineage was mostly responsible for outbreaks in the Indian subcontinent [12, 14, 214] and Southeast Asia [15, 215] (Fig. 3). The E1-K211E mutation first appeared in India in late 2009, as evidenced by isolate AP0109 (HM159390) from Hyderabad [216]. In 2010, a CHIKV strain harboring two novel mutations, E1: K211E and E2: V264A, was detected in all CHIKV isolates from New Delhi, India [44]. Furthermore, two new mutations, E1: K211E and E2: V264A, were identified in *Ae. aegypti-*dominated regions in Tamil Nadu [216] and Kolkata [217] in 2011 and 2012, respectively. An evolutionary analysis of the CHIKV ECSA lineage from 1953 to 2020 has been undertaken, and is shown in Fig. 4. The substitution of amino acid residue 211 of the E1 glycoprotein from lysine (K) to glutamic acid (E) is under positive selection pressure [216]. In contrast, no evidence has been found of positive selection for the substitution of amino acid residue 264 (V264A) of the E2 glycoprotein. The double-mutant virus containing E1: K211E and E2: V264A and lacking the A226V substitution enhanced the rates of infection, dissemination, and transmission of CHIKV in the *Ae. aegypti* vector when compared with those of CHIKV of the ECSA genotype with wild-type E1: 226A [218]. However, these double mutations did not have a significant effect on CHIKV fitness for *Ae. albopictus*. Here, we compile documents of CHIKV outbreaks in different countries of South Asia and Southeast Asia since 2015.

#### An outbreak in 2015–2017 in India

According to the National Vector Borne Disease Control Programme (NVBDCP), India reported 27,553 suspected cases and 3342 laboratory-confirmed cases of CHIKV infection in 2015. Most (75%) of the suspected cases of CHIKV infection were in Karnataka. The number of chikungunya cases continued to grow into 2016, when there were 64,057 suspected cases, 41.2% of which were diagnosed with CHIKV infection. A total of 55.4% of all the suspected cases and 53.9% of all the confirmed cases originated from three states in India, namely, New Delhi, Maharashtra, and Karnataka [219]. In 2017, a total of 67,769 suspected chikungunya cases were reported from states throughout the country. Remarkably, the *A. albopictus*-adaptive E1: A226V mutation, which has been responsible for the outbreaks in the Indian Ocean Islands and India since 2007 [28], was absent in recent Indian isolates. The 2015–2017 Indian isolates harbored *A. aegypti*-adaptive E1: K211E and E2: V264A mutations, in conjunction with wild-type E1: 226A. The current re-emergence of CHIKV strains possessing unique mutations in CHIKV E1, such as K16E/Q, K132Q/T, and S355T; and in CHIKV E2, such as C19R and S185Y, could be related to epitopes or virulence-determining domains [12]. During 2018–2020, India recorded more than 170,000 suspected and 27,120 confirmed cases in various states of the country [219].

#### An outbreak in 2017 in Bangladesh

From April 2017 to September 2017, Bangladesh experienced a significant outbreak of CHIKV, during which numerous chikungunya cases were documented in 23 districts [220]. Moreover, CHIKV rapidly spread throughout the capital city, Dhaka, which has more than 18 million inhabitants, resulting in over 13,000 clinically confirmed cases [221]. Although this recent outbreak in 2017 was driven by the CHIKV ECSA genotype, this isolate was genetically distant from the isolate responsible for the previous outbreak. Comprehensive phylogenetic analyses revealed that the strains from the 2017 outbreak formed a novel cluster within the ECSA with the 2016 South Asian strains, including the 2016 India isolate and the 2016 Pakistan isolate, suggesting that they represented a novel sub-lineage of the ECSA genotype [14]. The 2017 Bangladesh strains possessed two novel mutations, E1: K211E and E2: V264A, but lacked the E1: A226V substitution. Moreover, a novel I317V adaptive mutation identified in a 2017 Bangladesh isolate was also found in a southern Indian strain isolated during 2015–2016 [222] and a 2016–2017 strain isolated in the Central India [48].

#### An outbreak in 2016–2017 in Pakistan

Following the large CHIKV outbreak in India in 2016, a significant upsurge of CHIKV infection was documented in Karachi, which is adjacent to India; this spate started in November 2016, leading to over 30,000 suspected chikungunya cases, 4000 of which were laboratory confirmed [214]. Sequence analysis indicated that the current Pakistani strains lack the *Ae. albopictus*-adaptive E1: A226V and E2: K252Q mutations. Phylogenetic and migration analysis revealed that the 2016 outbreak in Pakistani occurred due to the importation of the virus strain from India [223]. A chikungunya outbreak occurred across the country from December 2016 to May 2017 [224]. The ECSA genotype caused the 2016–2017 outbreak in Pakistan. Whole-genome sequencing and phylogenetic analyses of CHIKV genotypes circulating in Pakistan during 2016–2017 revealed that the 2016–2017 Pakistan outbreak virus possessed two novel mutations, E1: K211E and E2: V264A, in the background of E1: 226A, which belongs to a cluster of novel ECSA [46, 212, 225].

#### An outbreak in 2018–2020 in Thailand

**Fig. 5:**
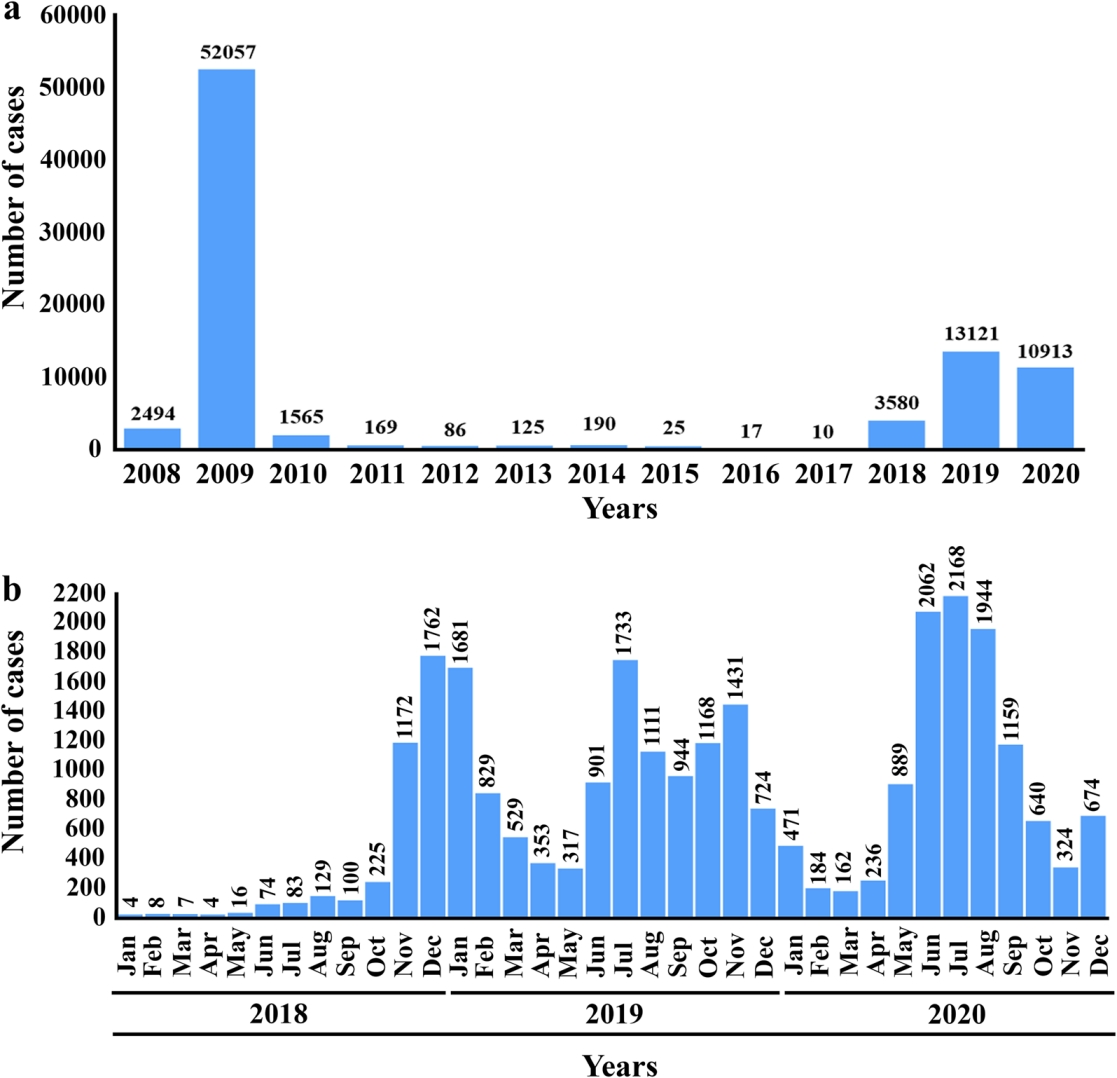
Chikungunya cases in Thailand. **a** The number of chikungunya cases per year between 2008 and 2020. **b** The monthly number of chikungunya cases in Thailand between 2018 and 2020

A second massive CHIKV outbreak started in late 2018 in the south of Thailand. The virus spread nationwide, and over 27,000 confirmed cases were documented between 2018 and 2020. According to the Bureau of Epidemiology, Ministry of Public Health, Thailand (Fig. 5) [186], monthly reported chikungunya cases started to rise in June 2018. All the initial cases were associated with individuals residing in Satun and Narathiwas provinces in the south of Thailand. The number of reported cases rose continuously from < 20 cases per month between January and May to 1171 and 1759 cases in November and December 2018, respectively. In 2019, chikungunya cases were reported in 60 provinces of the country, but most were from Bangkok and provinces in the south of Thailand. From January to November 2020, the outbreak was reported in 72 provinces across the country. Most of the cases of CHIKV infection were documented in Chanthaburi and several provinces in the north. Overall, chikungunya-associated morbidity rates in Thailand in 2018, 2019, and 2020 were 5.40, 19.73, and 15.69 per 100,000 population, respectively. The causative CHIKV strains of the second mass outbreak in Thailand belonged to the ECSA; however, these ECSA strains lacked the valine substitution at position 226 of CHIKV E1 and were grouped into a new distinct sub-lineage separate from ECSA-IOL CHIKV isolates of the previous outbreak during 2008–2009. The 2018–2020 Thai strains possesses two novel mutations, E1: K211E and E2: V264A, in the E1: 226A background [15, 47]. Genomic and phylogenetic analyses revealed that the 2018–2020 CHIKV strains isolated in Thailand were similar to the strains that caused the recent outbreak in South Asia during 2016–2017, but not to the causative agent of the first massive outbreak in the country that occurred in 2008–2009. The 2018–2020 Thai strains showed the greatest homology to sequences from Bangladesh, indicating that these viral strains were imported into Thailand from Bangladesh before becoming the local transmission cause [47].

#### An outbreak in 2019 in Myanmar

Following an outbreak of chikungunya in 2010 [190], no cases of CHIKV infection were officially documented in Myanmar until 2019. Recently, CHIKV outbreaks were reported in Kachin State, Nay Pyi Taw, Tanintharyi, and the Mandalay region [226]. The CHIKV from the 2019 outbreak in Myanmar was a new ECSA strain; this CHIKV strain lacked the E1: A226V substitution and harbored the E1: K211E mutation, whereas the ECSA-IOL with E1: 226V mutation caused the previous outbreak in Myanmar in 2010. Phylogenetic analysis of the partial E1 protein revealed that the present (2019) CHIKV strain in Myanmar was closely related to the strain circulating in Thailand in the same year [51].

## Conclusions

As shown in this brief review, CHIKV has emerged as a public health burden and has continued to circulate in South and Southeast Asia over the past decade. CHIKV of the ECSA-IOL genotype, harboring an *Ae. albopictus*-adaptive mutation (E1: A226V), has been responsible for the large CHIKV epidemics in Asia, especially in rural and suburban areas, since 2005. Recently, the emergence of a novel ECSA with *Ae. aegypti*-adaptive mutations (E1: K211E and E2: V264A, with E1: 226A) has been reported in many countries, starting around late 2009–2010 in India, which continued circulate and causing the massive outbreak in India in 2016, and Pakistan in 2016 before spreading to Bangladesh in 2017, Thailand in 2018, and Myanmar in 2019. The wide distribution of this novel ECSA carrying *Ae. aegypti*-adaptive mutations shows that CHIKV continues to spread at an alarming rate and can expand to new regions through the ever-increasing number of travelers in epidemic areas and adaptations of the strains to local mosquito vectors, particularly in urban areas. The differences between the CHIKV ECSA strains that caused the first mass outbreaks between 2005 and 2015 and the second ones from 2016 to 2020 in South and Southeast Asia suggest that molecular surveillance is necessary to determine the prevalence of viral strains and regional genotype differences, which may lead to a better understanding of virus adaptation, transmission, and outbreak tracking. Furthermore, data for CHIKV outbreak occurrence would aid in developing strategies for the control and prevention of CHIKV infection.

## Data Availability

Not applicable.

## Abbreviations

CHIKV: Chikungunya virus
ECSA-IOL: East/Central/South African strain-Indian Ocean lineage
